# A Case‐Control Investigation of Factors Associated With Risky Sexual Behaviors Among South African University Students

**DOI:** 10.1111/phn.70049

**Published:** 2025-12-02

**Authors:** Trishka Pillay, Nalini Govender, Poovendhree Reddy

**Affiliations:** ^1^ Department of Environmental Health Faculty of Applied and Health Sciences Mangosuthu University of Technology KwaZulu Natal South Africa; ^2^ Department of Community Health Studies Faculty of Health Sciences Durban University of Technology KwaZulu Natal South Africa; ^3^ Department of Basic Medical Science Faculty of Health Sciences Durban University of Technology KwaZulu Natal South Africa

**Keywords:** HIV, risk factors, risky sexual behavior, students, South Africa

## Abstract

**Objective:**

This study aimed to investigate risk factors associated with risky sexual behaviors (RSBs) and HIV seropositivity among students from four universities in KwaZulu‐Natal, South Africa.

**Design:**

A case‐control methodology was used.

**Sample:**

The sample consisted of 500 students (375 HIV negative students and 125 students living with HIV).

**Measurement:**

Adjusted logistic regression modeling was performed to assess the association of predictors with RSBs and HIV seropositivity.

**Results:**

Data showed that heavy episodic drinking [aOR: 2.73, (95% CI: 1.38; 5.44), *p* = 0.004], drugs before sex [aOR: 7.46, (95% CI: 2.11; 27.88), *p* = 0.003], and a higher number of lifetime sex partners increased students’ likelihood of having multiple concurrent sex partners (2–5 lifetime partners) [aOR: 4.22, (95% CI: 1.69; 10.54), *p* = 0.002] and ≥ 6 lifetime partners [aOR: 16.36, (95% CI: 6.18; 43.28), *p* < 0.001].

**Conclusion:**

These findings indicate a need for South African universities to offer HIV prevention programs that inform students of how participation in particular risky activities can result in engagement in specific RSBs, contributing to a heightened HIV infection risk. Re‐evaluation and strengthening of these prevention programs can ensure optimal efficiency in the battle against HIV infection.

## 1 Introduction

The global HIV prevalence data in 2021 confirmed that 38.4 million people were living with HIV, with a global HIV incidence of 1.5 million new cases (UNAIDS 2022). South Africa (SA) remains the epicenter for HIV infection, with an HIV prevalence of 7.7 million (Nuh 2021). The 2017 SA National HIV prevalence, incidence, behavior, and communication survey reported an overall HIV incidence of 231,100 new infections, with the highest incidence (88,400 new infections) among youth aged 15–24 years (Simbayi et al. 2019). Females within this age category appeared to be disproportionately affected by HIV in comparison to males (66,200 vs. 22,200) (Simbayi et al. 2019). In 2019, a 27.0% HIV prevalence was reported in the province of KwaZulu‐Natal (KZN) (Kharsany et al. 2019). Key drivers of this epidemic continue to include having multiple sexual partners, inconsistent condom usage, socioeconomic power imbalances, sexual violence, and substance abuse (Zuma et al. 2022). Economic factors viz., poverty were previously reported to influence women's participation in transactional sexual practices, affecting condom use negotiation and consequently increasing the risk of HIV infection (Whiteside 2010). Several interventions viz., the scale‐up of HIV treatment, youth focused interventions, and voluntary male medical circumcision (MMC), have been implemented thus far to counteract the rise in HIV infection (Nuh 2021). Despite these interventions, the number of new HIV infections continues to be a major public health concern.

Previous research specifically undertaken among vulnerable populations examined how risky activities such as alcohol and drug usage result in engagement in risky sexual behaviors (RSBs) among young people (Guo et al. 2002), men having sex with men (MSM) (Pufall et al. 2018) and university students (Mthembu et al. 2019). University students worldwide are considered vulnerable to RSB such as having multiple sexual partners (Hoque 2011), engaging in transactional sex (Masvawure 2010) and intergenerational sex (HEAIDS 2010), inconsistent condom use (Adam and Mutungi 2007), and unprotected vaginal sex (Fierros‐gonzalez and Brown 2002; Brown and Vanable 2007) predisposing them to HIV infection. An earlier study undertaken among students from 21 South African higher educational institutions (HEIs) reported an HIV prevalence of 3.4% [CI: 2.7%–4.4%] (HEAIDS 2010). Previous studies conducted in the sub‐Saharan African context, indicate that students’ susceptibility to RSBs stems from various behavioral and economic risk factors, including alcohol and drug use (Amare et al. 2019), peer pressure (Akintola et al. 2011), and a poor socio‐economic background (Ajayi and Somefun 2019). Despite several studies investigating risk factors associated with RSBs among South African university students (HEAIDS 2010; Mutinta 2014; Hoffman et al. 2017), the HEAIDS 2010 study is the only published HIV prevalence and risk factor stratification by HIV status report applicable to the South African university population. In view of this, more studies are required to investigate university students’ engagement in RSBs stratified by HIV status and the risk factors associated with HIV seropositivity as a means to develop effective HIV prevention programs. By developing an understanding of the extent to which students are currently engaging in RSBs, more targeted and evidence informed prevention programs can be developed to possibly curtail HIV incidence. It is important to understand and prevent students’ susceptibility to HIV infection given that this population group, particularly in low and middle‐income countries has an important role to play in leading and helping with the continued growth and development of their nations (Hoffman et al. 2017). The use of case‐control study designs investigating HIV status as an outcome of risky behaviors among MSM (Oster et al. 2011), circumcised men (Ediau et al. 2015), and patients that accessed voluntary counseling and testing (VCT) services (Carlos et al. 2017) has generated potentially useful data that can influence management. Since there is limited availability of case‐control data from the SA university context, this study investigated the behavioral risk factors associated with RSBs among students living with HIV and uninfected students from four public sector universities in KZN.

## 2 Materials and Methods

### 2.1 Design and Sample

Ethics approval was received from the Institutional Research Ethics Committee, Durban University of Technology, SA (IREC 005/19) and institutional approvals from all participating study sites. A total of 500 students were recruited between July 2019 and May 2021 via convenience sampling methods from four public HEIs in KZN, SA. Of this sample 375 comprised the control group (“HIV seronegative students”) and 125 were the cases (“students who were HIV seropositive”). All participants were registered students at participating HEIs and ≥ 18 years of age. The HIV status of participants was confirmed prior to participation in the study by VCT. Additionally, participants with a seronegative HIV status presented a negative HIV test to confirm their status. All participants who were HIV seropositive and had initiated antiretroviral treatment were briefed on the study by the respective nurses, and interested participants were referred to the study. Interested individuals who met the inclusion criteria were directed to the onsite research assistant based at a private location within the university health clinics. Those willing to participate were required to complete the informed consent form. Data were collected using a questionnaire with a unique identification number, thereby ensuring confidentiality.

### 2.2 Measures

The questionnaire included various variables adapted from previous validated questionnaires as follows: demographics including age category, sex and race, alcohol/ drug usage, heavy episodic drinking (Babor et al. 2001), sexual practices and engagement (Hoque 2011), contraceptive use (Hoque 2011; Hoque and Ghuman 2012a, 2012b), MMC (Leadbeatter 2012; Kharsany et al. 2015), sexually transmitted infection (STI) history, and peer influence (Bingenheimer et al. 2015).

Substance usage was assessed based on the following variables: alcohol use before sex in the past three months, drug use before sex in the past 3 months, and heavy episodic drinking. Heavy episodic drinking was evaluated by question 3 of the Alcohol Usage Disorders Identification Test (AUDIT) questionnaire, “How often do you have 6 or more drinks on one occasion?” (Babor et al. 2001). The response “never” was categorized as “non‐heavy episodic drinking” and “monthly or less, 2 to 4 times a month, 2 to 3 times a week, and 4 or more times a week” were categorized as ‘heavy episodic drinking’. Sexual practices and engagement were evaluated by variables such as age of sexual debut, multiple concurrent sex partners, lifetime number of sex partners, and condom usage. Multiple concurrent sex partners was defined as having more than one sexual partner that overlap in time (Mutinta 2014). Multiple concurrent sex partners were grouped into a binary “yes/no” response. Lifetime number of sexual partners was categorized as: 1 partner, 2 to 5 partners, and ≥ 6 partners. Age of sexual debut was dichotomized into < 15 and ≥ 15 years. Condom usage during vaginal sex was stratified into inconsistent and consistent usage. Reproductive health questions focused on use of contraceptives, type of contraceptives used, and reasons for not using contraceptives. The use of emergency contraception was also assessed.

### 2.3 Analytic Strategy

Data was exported into a Microsoft Excel spreadsheet and analyzed using Stata version 17 software. The chi‐square test was used to evaluate participant characteristics and reproductive health characteristics stratified by HIV status. Participant characteristics were stratified by multiple concurrent sexual partners among both case and control groups. Adjusted logistic regression modeling was performed to assess the association of predictors with RSBs and HIV seropositivity. In model 1, risk factors and demographics were tested to ascertain if they were determinants of RSBs. In the second model, risk factors and reproductive health characteristics were tested as possible predictors of HIV seropositivity. A confidence interval (CI) of 95% was used for all models and *p* < 0.05 was considered statistically significant.

## 3 Results

The participant characteristics stratified by HIV status is shown in Table 1. Of the 500 participants recruited, 32.7% were male and 67.3% were female; 60.2% comprised the 20–24 years age group; more than 75% were currently sexually active; 75.1% (244) reported no alcohol consumption and 91.1% (247) reported no drug usage before sex in the last 3 months (Table 1). Furthermore, 72.6% (143) of the HIV negative students reported engaging in heavy episodic drinking, and 10.1% (20) indicated drug use before sex; 5% (16) reported that the onset of sexual debut occurred at < 15 years; 48.7% (170) reported having sex with multiple concurrent sex partners; and 76.5% (195) indicated inconsistent condom usage. In contrast, the following statistics were reported by students living with HIV: heavy episodic drinking [29; 53.7%]; drugs before sex [4; 5.6%]; early sexual debut [1; 0.9%]; sex with multiple concurrent partners [34; 27.9%]; and inconsistent condom usage [70; 68%]). Heavy episodic drinking was significantly associated with an HIV negative status (*p* = 0.008). Moreover, a later age of sexual debut (≥18 years) was significantly associated with HIV positive status (*p* = 0.030). Notably, not having multiple concurrent sex partners was also significantly associated with an HIV positive status (*p* < 0.001).

**TABLE 1:** Participant characteristics stratified by HIV status.

Characteristics	HIV− (*N* = 375)	HIV+ (*N* = 125)
**Age** **		
Median (Q1, Q3)	22.0 (20.0, 24.0)	24.0 (22.0, 26.0)
**Age category (*n* = 498)** **		
<20	67 (18.0)	7 (5.6)
20–24	238 (63.8)	62 (49.6)
>24	68 (18.2)	56 (44.8)
**Number of lifetime sexual partners** **		
Median (Q1, Q3)	4.0 (2.0, 8.0)	3.0 (2.0, 5.0)
**Sex (*n* = 498)** **		
Male	146 (39.0)	17 (13.7)
Female	228 (61.0)	107 (86.3)
**Race (*n* = 496)**		
African	371 (100.0)	125 (100.0)
**Alcohol before sex (past 3 months) (*n* = 325)**		
No	192 (73.0)	52 (83.9)
Yes	71 (27.0)	10 (16.1)
**Heavy episodic drinking (*n* = 251)** *		
No	54 (27.4)	25 (46.3)
Yes	143 (72.6)	29 (53.7)
**Drug before sex (past 3 months) (*n* = 271)**		
No	179 (89.9)	68 (94.4)
Yes	20 (10.1)	4 (5.6)
**Currently sexually active (*n* = 472)**		
No	87 (24.7)	28 (23.3)
Yes	265 (75.3)	92 (76.7)
**Age of sexual debut (*n* = 437)** *		
<15 years	16 (5)	1 (0.9)
15–17 years	114 (35.6)	33 (28.2)
≥18 years	190 (59.4)	83 (70.9)
**Inconsistent condom use (*n* = 358)**		
Consistent	60 (23.5)	33 (32.0)
Inconsistent	195 (76.5)	70 (68)
**Multiple concurrent sex partners (*n* = 471)** **		
No	179 (51.3)	88 (72.1)
Yes	170 (48.7)	34 (27.9)

*p* < 0.05*, *p* < 0.005** was considered statistically significant

The behavioral risk factors stratified by multiple concurrent sexual partners are presented in Table 2. Being female was significantly associated with not having multiple concurrent sex partners among students living with HIV (*p* < 0.001). Moreover, a significant association was noted between being male and having multiple concurrent sex partners among the HIV negative group (*p* < 0.001). Heavy episodic drinking was significantly associated with having multiple concurrent sex partners among HIV negative participants (*p* = 0.029). Furthermore, a later onset (older age) of sexual debut (≥18 years) was significantly associated with not having multiple concurrent sex partners among both groups (*p* < 0.004). Most students reported not being pressurized to engage in sex; among the cases, not being pressurized by peers was significantly associated with not having multiple concurrent sex partners (*p* = 0.002).

**TABLE 2:** Behavioral characteristics stratified by multiple concurrent sex partners among participants living with HIV and uninfected participants (*N* = 500).

	Multiple partners HIV+		Multiple partners HIV–	
	No	Yes	No	Yes
	(*N* = 88)	(*N* = 34)	(*N* = 179)	(*N* = 170)
**Age**				
Median (Q1, Q3)	24.0 (22.0, 27.0)	24.0 (21.0, 26.0)	22.0 (20.0, 24.0)	22.0 (20.0, 24.0)
**Sex**				
Male	5 (5.7)	12 (35.3)**	38 (21.3)	99 (58.2)**
Female	82 (94.3)	22 (64.7)	140 (78.7)	71 (41.8)
**Alcohol before sex (past 3 months)**				
No	39 (88.6)	12 (70.6)	87 (77.7)	91 (66.9)
Yes	5 (11.4)	5 (29.4)	25 (22.3)	45 (33.1)
**Heavy episodic drinking**				
No	18 (51.4)	6 (33.3)	29 (36.3)	24 (21.8)*
Yes	17 (48.6)	12 (66.7)	51 (63.7)	86 (78.2)
**Drug before sex (past 3 months)**				
No	54 (100.0)	14 (77.8)**	73 (96.1)	95 (84.8)*
Yes	0 (0.0)	4 (22.2)	3 (3.9)	17 (15.2)
**Age of sexual debut**				
<15	1 (1.2)	0 (0.0)**	0 (0.0)	16 (9.9)**
15–17	17 (20.2)	16 (51.6)	35 (23.2)	77 (47.5)
≥18	66 (78.6)	15 (48.4)	116 (76.8)	69 (42.6)
**Peer pressure to have sex**				
No	86 (97.7)	27 (81.8)**	152 (85.4)	133 (78.2)
Yes	2 (2.3)	6 (18.2)	26 (14.6)	37 (21.8)

*Note*: Definitions: “Heavy Episodic drinking” is defined as: drinking 6 or more alcoholic drinks on one occasion (WHO, 2022).

*p < 0.05 and **p < 0.005 was considered statistically significant.

Table 3 illustrates the reproductive health status of the study population. Of those who responded, 78% of participants reported use of contraceptives, and contraceptive usage was significantly associated with being HIV positive (*p* < 0.001). Of note, 61.3% (245) of the students reported condoms as the most common contraceptive measure. Approximately 60% of the participants confirmed contraceptive use during their most recent sexual encounter. Contraceptive use during the most recent sexual encounter was significantly associated with being HIV positive (*p* < 0.001). Usage of emergency contraception (EC) was reported by 54.9% of the sample, and the most reported reason was due to condom breakage [45% among controls versus 61% among cases]; this was significantly associated with being HIV positive (*p* = 0.045). Many male participants reported being medically circumcised at a health care facility; being circumcised was significantly associated with being an HIV negative male (*p* = 0.009). Not being diagnosed with an STI was significantly associated with HIV negative status (*p* < 0.001).

**TABLE 3:** Reproductive health characteristics stratified by HIV status.

	HIV status	
	HIV−	HIV+
	(*N* = 375)	(*N* = 125)
**Contraceptive usage (*n* = 496)** **		
No	99 (26.6)	8 (6.5)
Yes	273 (73.4)	116 (93.5)
***Contraceptive type***		
**Condom (*n* = 400)**		
No	100 (35.7)	55 (45.8)
Yes	180 (64.3)	65 (54.2)
**Injection (*n* = 400)** **		
No	197 (70.4)	66 (55.0)
Yes	83 (29.6)	54 (45.0)
**Pills (*n* = 400)**		
No	245 (87.5)	110 (91.7)
Yes	35 (12.5)	10 (8.3)
**Loop (*n* = 400)** *		
No	276 (98.6)	112 (93.3)
Yes	4 (1.4)	8 (6.7)
**Hormonal patch (*n* = 400)**		
No	276 (98.6)	119 (99.2)
Yes	4 (1.4)	1 (0.8)
**Natural methods (*n* = 400)** *		
No	256 (91.4)	117 (97.5)
Yes	24 (8.6)	3 (2.5)
**Birth control implant (*n* = 400)**		
No	262 (93.6)	108 (90.0)
Yes	18 (6.4)	12 (10.0)
**Other (*n* = 397)**		
No	276 (99.3)	116 (97.5)
Yes	2 (0.7)	3 (2.5)
**Difficult to get contraceptives (*n* = 194)**		
No	142 (97.9)	46 (93.9)
Yes	3 (2.1)	3 (6.1)
***Reasons for not using contraceptives***		
**Condoms reduce sexual satisfaction (*n* = 194)**		
No	93 (64.1)	38 (77.6)
Yes	52 (35.9)	11 (22.4)
**For economic reasons (*n* = 194)**		
No	141 (97.2)	46 (93.9)
Yes	4 (2.8)	3 (6.1)
**Partner did not want it (*n* = 193)**		
No	136 (94.4)	45 (91.8)
Yes	8 (5.6)	4 (8.2)
**Knew nothing about contraception (*n* = 194)**		
No	142 (97.9)	46 (93.9)
Yes	3 (2.1)	3 (6.1)
**In love with partner (*n* = 194)** *		
No	115 (79.3)	45 (91.8)
Yes	30 (20.7)	4 (8.2)
**Fear of contraception causing sterility (*n* = 194)**		
No	110 (76.4)	42 (84.0)
Yes	34 (23.6)	8 (16.0)
**Unavailability of contraception (*n* = 193)**		
No	131 (91.6)	48 (96.0)
Yes	12 (8.4)	2 (4.0)
**Did not think of contraception at that time (*n* = 194)**		
No	129 (89.6)	42 (84.0)
Yes	15 (10.4)	8 (16.0)
**Other (*n* = 194)**		
No	123 (86.0)	41 (80.4)
Yes	20 (14.0)	10 (19.6)
**Contraceptive usage at last sex (*n* = 465)** **		
No	153 (44.3)	30 (25.0)
Yes	192 (55.7)	90 (75.0)
**Did your partner use any form of contraception the last time you had sex?** ** **(*n* = 456)**		
No	180 (53.4)	45 (37.8)
Yes	157 (46.6)	74 (62.2)
***Emergency Contraception (EC)***		
**Have you or your partner ever used emergency contraception EC (*n* = 481)**		
No	154 (42.8)	63 (52.1)
Yes	206 (57.2)	58 (47.9)
**Reasons for using emergency contraception (*n* = 271)** *		
Condom broke	95 (45.5)	38 (61.3)
I/ my partner forgot to take birth control pills at that time	14 (6.7)	2 (3.2)
We had so much to think about, we didn't even think about contraception	52 (24.9)	5 (8.1)
I normally take emergency contraception	25 (12.0)	8 (12.9)
My partner forced me to have sex	2 (1.0)	0 (0.0)
Other	21 (10.0)	9 (14.5)
***Male Circumcision***		
**Circumcised (*n* = 163)** *		
No	26 (17.9)	8 (44.4)
Yes	119 (82.1)	10 (55.6)
**If so, have you undergone medical circumcision in a clinic/hospital (*n* = 129)**		
No	13 (10.9)	0 (0.0)
Yes	106 (89.1)	10 (100.0)
**STI diagnosis (*n* = 498)** **		
No	306 (81.8)	76 (61.3)
Yes	68 (18.2)	48 (38.7)

Abbreviation: STI, sexually transmitted infection (Table 3).

*p < 0.05 and **p < 0.005 was considered statistically significant.

The adjusted logistic regression modeling for risk factors, demographics, and RSBs is shown in Table 4. Participants who used drugs prior to sex were 7.46 more times more likely to have multiple sex partners [aOR: 7.46 (95% CI: 2.00; 27.88), *p* = 0.003]. Heavy episodic drinking was significantly associated with a 2.73 times higher likelihood of having multiple sex partners [aOR: 2.73 (95% CI: 1.38; 5.44), *p* = 0.004]. An older age of sexual debut (≥15 years old) was identified as a protective factor from having multiple sexual partners [aOR: 0.08 (95% CI: 0.01; 0.63), *p* = 0.017]. Respondents who had two to five lifetime sexual partners were 4.22 times more likely to have multiple sex partners [aOR: 4.22 (95% CI: 1.69; 10.54), *p* = 0.002]. Moreover, those who had ≥ 6 lifetime partners were at a 16.36 times higher risk of having multiple sex partners [aOR: 16.36 (95% CI: 6.18; 43.28), *p* < 0.001]. Male participants were at a 5.73 times higher risk of having multiple sex partners in comparison to female students [aOR: 5.73 (95% CI: 3.48; 9.44), *p* < 0.001]. Furthermore, males were 4.66 times more likely to have ≥ 2 lifetime sexual partners [aOR: 4.66 (95% CI: 1.91; 11.36), *p* = 0.001].

**TABLE 4:** Model 1 adjusted models for risk factors, demographics, and risky sexual behavior (*N* = 500).

			Multiple concurrent sex partners	Inconsistent condom use
Risk factors	Controls, *n* (%)	Cases, *n* (%)	AOR 95% CI	AOR 95% CI
**Alcohol use before sex (past 3 months)**				
No	192 (73)	52 (83.9)	1.00^1a^	1.00^1b^
Yes	71 (27)	10 (16.1)	1.58 [0.87; 2.86]	1.03 [0.53; 2.00]
**Heavy episodic drinking**				
No	54 (27.4)	25 (46.3)	1.00^2a^	1.00^2b^
Yes	143 (72.6)	29 (53.7)	2.73 [1.38; 5.44]**	0.60 [0.26; 1.38]
**Drug use before sex (past 3 months)**				
No	179 (89.9)	68 (94.4)	1.00^3a^	1.00^3b^
Yes	20 (10.1)	4 (5.6)	7.46 [2.00; 27.88]**	2.79 [0.61; 12.77]
**Age of sexual debut**				
<15 years old	16 (5)	1 (0.9)	1.00^4a^	1.00^4b^
≥15 years old	304 (95)	116 (99.1)	0.08 [0.01; 0.63]*	0.23[0.03; 1.85]
**Lifetime no. of sexual partners**				
1	41 (14)	13 (11.7)	1.00^5a^	1.00^5b^
2–5	145 (49.7)	76 (68.5)	4.22 [1.69; 10.54]**	0.94 [0.39; 2.22]
≥6	106 (36.3)	22 (19.8)	16.36 [6.18; 43.28]**	0.70 [0.27;1.82]

			Multiple partners	Lifetime no of partners
**Demographics**	Controls, *n* (%)	Cases, *n* (%)	AOR 95% CI	AOR 95% CI
**Sex**				
Female	228 (61)	107 (86.3)	1.00 ^6a^	1.00 ^6b^
Male	146 (39)	17 (13.7)	5.73 [3.48; 9.44]**	4.66 [1.91; 11.36]**
**Age category**				
<20 years	67 (18)	7 (5.6)	1.00 ^7a^	1.00 ^7b^
20–24 years	238 (63.8)	62 (49.6)	0.93 [0.45; 1.93]	1.34 [0.60; 2.99]
>24 years	68 (18.2)	56 (44.8)	1.52 [0.62; 3.73]	2.01 [0.74; 5.46]

*Note*: Adjusted for: 1a sex, food insecurity & HIV status; 1b sex & HIV status; 2a age category, sex, food security, HIV status; 2b age category, sex, HIV status; 3a,b sex & HIV status; 4a sex, food insecurity & HIV status; 4b sex & HIV status, 5a,b sex & HIV status; 6a adjusted for age category, age of sexual debut, food insecurity and HIV status; 6b adjusted for age category & HIV status; 7a adjusted for food insecurity, relationship status, alcohol before sex and HIV status; 7b adjusted for sex & HIV status.

*p < 0.05 and **p < 0.005 was considered statistically significant.

The adjusted logistic regression modeling for risk factors, reproductive health, and HIV seropositivity data is presented in Table 5. Participants who reported being diagnosed with an STI were at 2.46 times higher risk of HIV seropositivity [aOR: 2.46, (95% CI: 1.50; 4.03), *p* < 0.001]. The use of emergency contraception was protective against HIV seropositivity [aOR: 0.57, (95% CI: 0.36; 0.90), *p* = 0.016]. Furthermore, approximately 54.8% of participants reported having two to five lifetime partners and 31.8% had ≥ 6 lifetime partners. Inconsistent condom use was a protective factor against HIV seropositivity in this sample [aOR: 0.55, (95% CI: 0.31; 0.97), *p* = 0.037].

**TABLE 5:** Model 2 adjusted models for risk factors, reproductive health, and HIV seropositivity (*N* = 500).

			HIV seropositivity
Risk factors	Controls, *n* (%)	Cases, *n* (%)	AOR 95% CI
**Alcohol use before sex (past 3 months)**			
No	192 (73)	52 (83.9)	1.00^7a^
Yes	71 (27)	10 (16.1)	0.63 [0.29; 1.37]
**Drug use before sex (past 3 months)**			
No	179 (89.9)	68 (94.4)	1.00^7b^
Yes	20 (10.1)	4 (5.6)	0.71 [0.22; 2.33]
**Lifetime no. of sexual partners**			
1	41 (14)	13 (11.7)	1.00^7c^
2–5	145 (49.7)	76 (68.5)	1.62 [0.79;3.32]
≥6	106 (36.3)	22 (19.8)	1.02 [0.44;2.38]
**Multiple partners**			
No	179 (51.3)	88 (72.1)	1.00^7d^
Yes	170 (48.7)	34 (27.9)	0.64 [0.38; 1.06]
**Usage of emergency contraception**			
No	154 (42.8)	63 (52.1)	1.00^7e^
Yes	206 (57.2)	58 (47.9)	0.57 [0.36; 0.90]*
**STI diagnosis**			
No	306 (81.8)	76 (61.3)	1.00^7f^
Yes	68 (18.2)	48 (38.7)	2.46 [1.50; 4.03]**
**Inconsistent condom use (vaginal)**			
Consistent	60 (23.5)	33 (32.0)	1.00^7g^
Inconsistent	195 (76.5)	70 (68)	0.55 [0.31; 0.97]*

*p < 0.05 and **p < 0.005 was considered statistically significant. Adjusted for 7a, b, c, d, e, f, g sex & age category.

## 4 Discussion

Our data suggests that high‐risk activities, like substance use, are associated with engagement in RSBs among university students in KZN, SA. Heavy episodic drinking (HED) and drug usage were significantly associated with having multiple concurrent sexual partners. In contrast, a later age of sexual debut was protective against multiple concurrent sex partners. By using a case‐control design, we found that a greater proportion of HIV negative participants engaged in these high‐risk activities, predisposing them to RSBs, such as inconsistent condom use and having multiple concurrent sexual partners. University students who participate in high‐risk activities are more likely to engage in RSBs, thereby predisposing themselves to an increased risk of HIV infection and unplanned pregnancy.

Notably, HED was more commonly reported among the HIV uninfected than students living with HIV (143; 72.6% vs. 29; 53.7%). Our data contradicts findings from Ediau et al. (2015), who reported that 60% of people living with HIV (*n* = 21) consumed alcohol prior to sex in comparison to 40% of HIV uninfected individuals (14) (Ediau et al. 2015). Heavy episodic drinking is a concerning issue among SA university students, as 14% of 837 university students reported being engaged in HED (Nkoana et al. 2016). Almost 69% of all students in our study reported engaging in HED, which is alarming since these students were 2.73 times more likely to have multiple concurrent sex partners compared to those who did not binge drink. Furthermore, among the HIV negative participants, HED was significantly associated with having multiple concurrent sex partners (*p* = 0.029). Excessive alcohol consumption has been previously reported as a determinant of high‐risk sexual behavior among young people (Guo et al. 2002). This risky practice may negatively impact the consistent and effective usage of condoms among this cohort thus compromising HIV prevention methods (Mthembu et al. 2019). Our data suggests that HED may encourage student engagement in RSBs (i.e., multiple concurrent sex partners), a practice that may potentially increase the risk of acquiring HIV among the HIV uninfected students. However, findings from the HEAIDS (2010) study revealed that students who reported being drunk once in the previous month were significantly less likely to be HIV positive (HEAIDS 2010). The university environment creates a social and cultural atmosphere that is prone to peer pressure and consequent substance use and abuse (Tayob and Van Der Heever 2014). Binge drinking together with sexual activities are reported as a source of recreation among university students thereby promoting casual sexual encounters (HEAIDS 2010). Notwithstanding various HIV prevention efforts targeted at students registered at HEIs, HIV uninfected students are continuing to engage in risky activities like HED, which may increase their HIV infection risk.

In our study, more controls (HIV negative participants) reported using drugs before sex in comparison to the cases (10.1% vs. 5.6%). Those who used drugs prior to sex were at a 7.46 times higher risk of having multiple concurrent sex partners. However, this finding must be interpreted with caution since only 8% (24) of the sample reported using drugs before sex. Our low prevalence drug use data is consistent with findings from the HEAIDS study, which confirmed that only 1% of their cohort (i.e., student, administrative and service staff) reported the use of injectable drugs. Alcohol and drug usage are reported as significant predictors of RSBs viz., inconsistent condom use and multiple concurrent sexual partners (Ross et al. 2001; Jones et al. 2011; Choudhry et al. 2014; Amare et al. 2019). A meta‐analysis study on substance use and sexual behavior in Peru confirmed higher rates of substance use among populations living with HIV compared to the general population (Massa and Rosen 2012). Drug use has been associated with a higher prevalence of HIV seropositivity (Massa and Rosen 2012), indicative that substance usage is an important predictor of RSBs. In contrast, there was no statistically significant association between usage of injectable drugs in the past month and HIV infection (HEAIDS 2010). Substance usage, combined with peer pressure and new‐found student independence, are reported as possible predictors for students engaging in RSBs (Tesfaye et al. 2014). A South African study conducted in 2012 among university students found that the likelihood of a student using drugs was 2.6 times higher in the sexually active group in comparison to sexually inactive students (Blignaut et al. 2015). Alcohol or drug use may impair students’ decision‐making processes, consequently limiting their ability to negotiate condom usage, which may increase their risk of HIV infection and unplanned pregnancy (Blignaut et al. 2015). Participants living with HIV in our study appeared to be more cautious in their behavior compared to the HIV negative participants. Despite the exposure to education and other HIV interventions, the HIV negative participants increased their risk of HIV infection as a result of substance usage and HED.

Our data confirm a similar sexual debut age for both controls and cases (≥15 years of age; 95% controls and 99.1% cases). Results found that a later age of sexual debut was significantly associated with a positive HIV status. This is contrary to previous reports (Bassols et al. 2010; Wand and Ramjee 2012), which indicate that an HIV positive status was significantly associated with earlier age of sexual debut. In our sample, the association between a later age of sexual debut and HIV seropositivity could be due to participants debuting sex with older partners who were living with HIV. The age of the sexual partner may be a risk factor for HIV infection independent of an individual's own age (Akullian et al. 2017). Moreover, having an older male partner or engaging in transactional sex with a partner who is HIV positive increases the risk of infection regardless of the age of sexual debut. In our study, those students who had a later age of sexual debut (≥15 years of age) were protected from having multiple concurrent sex partners [aOR: 0.08, (95% CI: 0.01; 0.63), *p* = 0.017]. Our finding corroborates that of Raj et al. (2017), who concluded that for every year of delay in sexual debut, the risk of STI decreased by 17% [OR: 0.83, (95% CI: 0.74; 0.93), *p* = 0.001]. Additionally, an early age of sexual debut was previously reported as a risk factor for multiple concurrent sex partners (Mah (2010), and may be associated with an increased risk for STI and HIV transmission (Manhart et al. 2002). In our study, a larger proportion of students living with HIV reported between 2 to 5 lifetime sex partners compared to HIV uninfected students, which contradicts findings from Carlos et al. (2017). These researchers reported finding similarities regarding ≥ 2 lifetime sexual partners in both cases and controls (89.78% vs. 83.51%) (Carlos et al. 2017). Our findings indicated that students who had more than 6 lifetime sexual partners were more likely to have multiple concurrent sex partners than those who had a single lifetime partner. These findings corroborate those of Mah (2010), who reported that individuals with ≥ 5 lifetime partners were more likely to report having concurrent sexual partners compared to those with 1 to 3 lifetime partners. Kogan et al. (2015) suggested that among young African American men, having multiple concurrent sex partners was significantly associated with other RSBs such as inconsistent condom use and self‐reported STIs. These findings imply that having more than one lifetime partner increases the risk of RSB engagement and an early sexual debut directly influences the number of future sexual partners, which may perpetuate the spread of HIV infection.

Our findings highlight condom use as a preferred contraceptive method (61.3%), even though 74% of participants reported inconsistently using condoms during sexual activity. These findings are consistent with data from Turkey (Bal Yilmaz et al. 2010) and Tanzania (Sweya et al. 2016). Alarmingly, inconsistent condom use was higher among the HIV negative group compared to cases (controls 195; 76.5% vs. cases 70; 68%). Similar data emerged from a case control study among STI participants, which revealed a greater proportion of STI uninfected participants engaged in unprotected sex in comparison to STI infected participants (controls 91.1% vs. cases 72.3%) (Raj et al. 2017). Despite a higher prevalence of inconsistent condom use among controls than cases, 68% of students living with HIV reported inconsistent use of condoms, consequently increasing the risk of HIV infection among their sexual partners. Regular HIV VCT is thus recommended in HEIs, which increases the likelihood of early detection of HIV and prompt initiation of ARV treatment in affected students. Consequently, this will reduce viral loads to further prevent the transmission of HIV and optimize student health. Moreover, regular VCT is required for HIV negative students who are in serodiscordant relationships and timely initiation of Pre‐exposure prophylaxis (PrEP) should be introduced to prevent infection.

Protection against HIV, STIs, and pregnancy are dependent on the effective and consistent usage of condoms (Simbayi et al. 2019). Interestingly, Mthembu et al. (2019) found that consistent condom use was practiced among students who perceived themselves to be at an increased risk of unplanned pregnancies and HIV (Mthembu et al. 2019). In our study, 34% (63) of the respondents reported not using contraceptives such as condoms as they reduced sexual satisfaction. Only 26% reported consistent use of condoms during intercourse, with a greater proportion of cases reporting consistent condom use in comparison to the controls. This is suggestive that participants living with HIV were being more responsible with their sexual health in comparison to the HIV negative participants. These individuals may have wanted to protect themselves from STIs and unwanted pregnancy. Furthermore, participants living with HIV may have been more health conscious due to awareness of their status and opted to use condoms to protect their partner from HIV infection. Inconsistent use of condoms among SA youth may be attributed to various issues, including difficulties experienced when negotiating condom use with male partners, stable relationship status, alcohol use and/or abuse, and the perceived poor quality and/or dissatisfaction experienced with freely available condoms (Mthembu et al. 2019; Simbayi et al. 2019). Inconsistent condom use may unfortunately lead to STIs. In our study, the diagnosis of an STI increased the risk of HIV seropositivity by 2.46 times, a finding consistent with a previous report (Abdool Karim and Abdool Karim 2010) which highlighted the increased probability of HIV infection among individuals with STIs. However, there is a possibility of other factors not evaluated in this study, such as perinatal infection, and the use of contaminated needles during injectable drug usage, etc., which may have contributed to the HIV seropositivity. Future studies are required to further evaluate this association. Current condom promotion interventions need to be re‐evaluated and improved to increase students’ consistent condom usage. Considering the increased freedom and peer pressure experienced by first time entry university students, special education efforts should be targeted and tailored to their unique needs.

In sub‐Saharan Africa, MMC significantly reduces the risk of HIV infection in young men (Auvert et al. 2005; Gray et al. 2007). In our study, a high percentage of both the cases and controls reported undertaking medical circumcision in a clinical setting (89.1%; 106 controls vs. 100%; 10 cases). In Uganda, only 8.4% of cases and 21.9% of controls were medically circumcised from a cohort of 155 men living with HIV and 155 HIV uninfected men (Ediau et al. 2015). Knowledge and awareness of the benefits of MMC is integral in uptake of this practice. Moreover, students who perceived that they were at high risk of HIV infection were 1.28 times more likely to be circumcised; however, understanding the effectiveness of MMC as an HIV prevention strategy positively increases its uptake (Mamo et al. 2018). In contrast, a Nigerian study revealed that only 38% (*n* = 144) of their student cohort were knowledgeable that MMC may reduce their risk of HIV infection (Iliyasu et al. 2012). Our data is indicative of an increased knowledge and awareness among students regarding the benefits associated with MMC in the prevention of HIV. Increased health promotion efforts on MMC among students can be beneficial in the uptake of these services.

Findings from this study reveal the urgent need for a comprehensive HIV prevention strategy to successfully reduce infections among South African youth. Ideally, this strategy must incorporate biomedical, behavioral, and structural interventions with psychosocial support and educational outreach programs. Successful implementation requires identifying and addressing context‐specific barriers, including stigma, knowledge gaps, and socio‐economic vulnerabilities that restrict access to and utilization of HIV prevention services. Addressing these barriers will ultimately enhance the uptake of HIV prevention interventions. Targeted educational interventions that address the risks associated with substance use may further promote safer sexual practices and contribute to lower HIV transmission rates. This multipronged strategy holds the potential to alleviate strain on the national healthcare system, enhance economic productivity, and safeguard the health and future of young South Africans.

### 4.1 Limitations and Future Research

A possible limitation of this study is the use of self‐reported data collection tools, which could be subject to recall bias. Furthermore, all participants belonged to one racial group and most study participants were female. Future large‐scale cohort studies are required to investigate exposures such as RSBs and HIV seropositivity among university students from enrolment to exit to determine causal associations.

### 4.2 Recommendations

Campus substance use prevention programs are required to identify students at risk of HED and drug abuse to ensure interventions are better targeted to address the specified ways in which they are engaging in risky activities. For instance, in terms of education, our findings indicated that HIV uninfected students must be sensitized about how irresponsible drinking and drug use can lead to engagement in RSBs. In addition, in terms of prevention strategies, HIV uninfected students with elevated risk levels may benefit from use of PrEP to prevent HIV infection. Regarding age of sexual debut, the re‐evaluation and strengthening of reproductive health education at a primary school level must be considered to encourage an older age of sexual debut, which has a potential to prevent an increase in the number of subsequent lifetime sexual partners. These efforts should provide education on delaying sexual debut as well as the importance of correct and consistent condom usage to prevent HIV and unwanted pregnancy.

Programs strengthening condom use efficiency and consistency, as well as improving condom negotiation skills, must be strengthened for both students living with HIV and uninfected students. Health promotion efforts on preventing HIV transmission should be intensified among students living with HIV. Additionally, health promotion must be strengthened on educating students about the benefits of MMC in HIV prevention to further improve uptake of services. The promotion of MMC among HIV uninfected students is necessary to help curb HIV infection among these students.

### 4.3 Implications for Public Health Nursing

Nurses play an indispensable role in HIV prevention, in particular within the university health clinic context. The role of nurses includes VCT services and health education on HIV preventative strategies, that is, PrEP and condom usage. Furthermore, nurses encourage healthy lifestyle practices such as safe sexual practices, avoidance of substance usage and regular HIV screening. Clinical assessments of students for HIV prevention strategies such as PrEP should consider engagement in risky activities, that is, substance usage which may subsequently heighten the HIV infection risk among students. Nurses also play a crucial role in the referral of affected students to university substance usage programs for further intervention.

## 5 Conclusion

To our knowledge, the HEAIDS report is the only data available that investigated this association within this high‐risk population. The HEAIDS findings are more than a decade old and thus we provide an update in the literature regarding the current behaviors of this vulnerable population, which may be used to inform HIV prevention interventions and policymakers. The findings from our study are novel, as they highlight HIV negative participants as a more vulnerable group to engage in risky activities and RSBs in comparison to participants living with HIV in HEIs. The data suggest that students living with HIV were more health‐conscious in comparison to the HIV uninfected students. Furthermore, this study investigated the association between risky activities and/or RSBs and HIV seropositivity among a South African university population, who are considered high risk. Our findings suggest that HED and drug usage should be considered potential predictors of RSBs among a SA university student population. Educational programs on prevention methods, such as the correct and consistent use of condoms, require strengthening; consequently, this will aid in preventing HIV infection and further transmission. Despite many HIV prevention programs and health education efforts, students continue to engage in RSBs, particularly the HIV uninfected student population. University students should be identified as a vulnerable population and must be considered when developing interventional tools regarding substance use education and RSB programs. This population requires guidance regarding the risks of engaging in risky activities such as HED and drug usage, and consequent predisposition to RSBs.

## Ethics Statement

Ethics approval for this study was granted by the Institutional Research Ethics Committee, Durban University of Technology, South Africa (IREC 005/19).

## Conflicts of Interest

The authors declare no conflicts of interest.

## Data Availability

Data supporting the results of this study are available upon request from the corresponding author. However, data cannot be used for purposes other than data confirmation. Due to privacy or ethical restrictions data are not publicly available.
